# Methods for negating the impact of zinc contamination to allow characterization of positive allosteric modulators of glycine receptors

**DOI:** 10.3389/fnmol.2024.1392715

**Published:** 2024-06-24

**Authors:** Casey I. Gallagher, David P. Bishop, Thomas E. Lockwood, Tristan Rawling, Robert J. Vandenberg

**Affiliations:** ^1^Discipline of Pharmacology, School of Medical Sciences, University of Sydney, Sydney, NSW, Australia; ^2^Hyphenated Mass Spectrometry Laboratory, Faculty of Science, University of Technology Sydney, Broadway, NSW, Australia; ^3^School of Mathematical and Physical Sciences, Faculty of Science, The University of Technology Sydney, Sydney, NSW, Australia

**Keywords:** zinc, glycine receptor, chelator, tricine, positive allosteric modulator, bioactive lipid, drug development

## Abstract

Zinc is a ubiquitous contaminant in many buffers, purified products and common labware that has previously been suggested to impact on the results of functional GlyR studies and may inadvertently cause the effectiveness of some GlyR modulators to be over-estimated. This could greatly impact the assessment of potential drug-candidates and contribute to the reduced effectiveness of compounds that reach clinical stages. This is especially true for GlyR modulators being developed for pain therapeutics due to the changes in spinal zinc concentrations that have been observed during chronic pain conditions. In this study we use two-electrode voltage clamp electrophysiology to evaluate the metal chelators tricine and Ca-EDTA, and show that tricine produces inhibitory effects at GlyRα_1_ that are not mediated by zinc. We also utilized the zinc insensitive W170S mutation as a tool to validate metal chelators and confirm that zinc contamination has not impacted the examination of lipid modulators previously developed by our lab. This study helps to further develop methods to negate the impact of contaminating zinc in functional studies of GlyRs which should be incorporated into future studies that seek to characterize the activity of novel modulators at GlyRs.

## 1 Introduction

Zinc is a divalent metal cation that is ubiquitous throughout nature. It is the second most abundant micronutrient in living organisms following iron, and the second most abundant divalent cation after calcium (Chasapis et al., 2020; Maares and Haase, 2020). This causes zinc to be an important mediator of a range of physiological functions. Bioinformatic approaches suggest that zinc can bind to over 2,800 proteins within the human genome (Andreini et al., 2006) including those required for gene expression, neurotransmission, and cellular processes such as proliferation and cell signaling [For reviews see Maares and Haase (2020) and Blakemore and Trombley (2017)]. Within the human body zinc is predominantly distributed within the bone and muscle, accounting for up to 87% of the total body concentration. However, recently an abundance of research has highlighted its importance as a neuromodulator at synapses throughout the CNS (Frederickson et al., 2000; Wang et al., 2023). A primary example of this is zinc modulation of glycine receptors (GlyRs).

GlyRs are pentameric ligand gated ion channels (pLGICs) that mediate inhibitory neurotransmission in the central nervous system. They belong to a family of membrane receptors known as the cys-loop receptors, which also contains the excitatory nicotinic and serotonergic gated ion channels, as well as the inhibitory GABA_A_ receptor. GlyRs are expressed throughout the CNS, including the spinal cord where they mediate sensory and nociceptive signaling (Takazawa and MacDermott, 2010; Lu et al., 2013). These receptors are primarily composed of the α_1_ and α_3_ subunits (San Martín et al., 2022) – which make GlyRα_1_ and GlyRα_3_ desirable candidates for drug-development for pain therapeutics (Cioffi, 2018; Gallagher et al., 2022).

Zinc has been shown to co-localize within glycinergic vesicles present at nociceptive synapses (Birinyi et al., 2001) where it mediates neuronal excitability by binding directly to GlyRs (Laube, 2002; Zhang and Thio, 2007; Perez-Rosello et al., 2015). The tonic concentration of zinc in the extracellular space is estimated to be in the low nanomolar range (Frederickson et al., 2006) but can reach synaptic concentrations of at least 10 μM during activation (Zhang et al., 2016). Within this physiological concentration range, zinc can endogenously modulate synaptic and non-synaptic GlyRs, and has also been shown to act pre-synaptically to facilitate glycine release (Birinyi et al., 2001). Endogenous levels of zinc enhance tonic GlyR activity, which significantly increases the mean amplitude of miniature Inhibitory Postsynaptic Currents (mIPSCs) reduces decay kinetics in spinal neurons (Laube, 2002) and decreases neuronal excitability within hippocampal neurons (Zhang and Thio, 2007) and auditory nerves (Perez-Rosello et al., 2015). This incurs a protective role by preventing overaction of the auditory system to prevent tinnitus (Zhang and Thio, 2007). Furthermore, in neuropathic pain models a reduction in vesicular zinc concentrations within the dorsal horn of the spinal is observed, and this directly correlates to the lowering of pain thresholds (Jo et al., 2008).

A pathological missense mutation in the GLRA1 gene that impacts zinc modulation has been found in hyperekplexia patients (Al-Futaisi et al., 2012). This produces GlyRα_1_ receptors with a W170S mutation that abolishes zinc potentiation without disrupting intrinsic receptor properties (Zhou et al., 2013). Some groups have suggested this mutation induces hyperekplexia by impacting on synaptic clustering when expressed in artificial synapses (Zhang et al., 2016). However, when spinal cords from a W170S hyperekplexic mice line were examined, the mutation was not found to impact on intrinsic receptor functioning or ligand binding properties, and the spinal cords were shown to have normal receptor expression levels and synaptic clustering *in vivo* compared to the spinal cords of control animals (McCracken et al., 2013a). These findings demonstrate that removing positive zinc modulation of GlyRs is sufficient to produce a hyperekplexia phenotype, even while retaining normal GlyR function and expression. Furthermore, this suggests that endogenous zinc concentrations are not only sufficient to modulate GlyRs *in vivo,* but are essential to maintain normal glycinergic neurotransmission throughout the nervous system.

**Figure 1:**
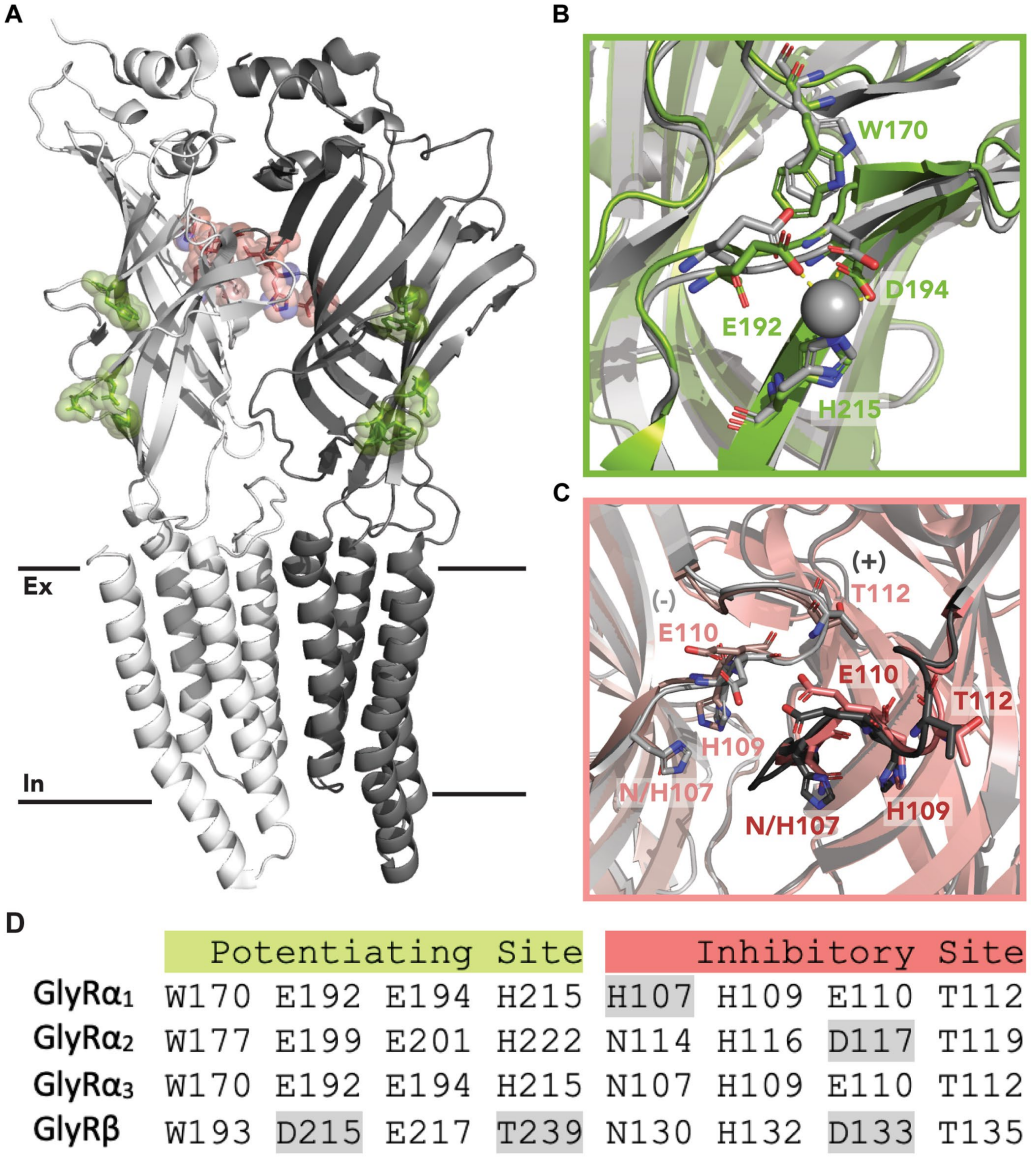
Zinc binding sites in GlyRs. **(A)** Location of the proposed inhibitory (red) and potentiating (green) zinc binding sites within the ECD of GlyRs. The primary subunit is shown in white, and the complementary subunit is shown in gray. Black lines indicate the lipid membrane with the extracellular (Ex) and intracellular (In) leaflets labeled. **(B)** Potentiating zinc binding cavity of GlyRα_3_ with co-ordinating residues shown in green. A zinc ion is shown in gray, and is coordinated by E192, D194 and H215. An aligned structure of GlyRα_1_ with coordinating residues is shown in gray. **(C)** Proposed inhibitory binding site of GlyRα_3_ viewed from the center of the receptor with important residues shown in red from the primary (dark) and complementary (light) subunit. An aligned structure of GlyRα_1_ with coordinating residues is shown in gray. **(D)** Conservation of zinc binding residues in GlyR subunits. Non-conserved residues are highlighted in gray. Accession code for PBD file used: 5TIO (GlyRα_3_) and 8DN4 (GlyRα_1_).

Zinc modulates GlyR activity in a biphasic manner; producing dose-dependent potentiation between 10 nM - 10 μM and inhibition at concentrations above 100 μM (Bloomenthal et al., 1994; Lynch et al., 1998; Harvey et al., 1999; Miller et al., 2005). This occurs through two distinct binding sites. The first is a low-affinity, inhibitory site that occurs at α-α or α-β interfaces within the extracellular domain (ECD) (Harvey et al., 1999; Nevin et al., 2003). A crystal structure of GlyRα_1_ shows a single zinc ion which is coordinated by residues H107 and H109 from the principal subunit, and E110 and T112 from the complementary subunit (Figure 1). Mutation of these residues significantly reduces or abolishes zinc inhibition without affecting zinc potentiation (Laube et al., 2000). The second site is a high affinity, potentiating site that occurs at an intra-subunit binding cavity within the ECD (Figure 1) (Huang et al., 2017). Zinc ions at this site are coordinated by E192 and D194 from the β9-strand, and H215 from loop C. Mutating these residues to alanine significantly reduces or abolishes zinc potentiation of GlyRα_1_ (Miller et al., 2005). Differences in these residues between receptor subunits also accounts for the differing zinc sensitivity between GlyRs (Hsiao et al., 2001). Intracellular binding sites have also been proposed from functional studies which identified a slowly reversible inhibitory component of zinc and a potentiation effect that occurred when zinc was applied via an intracellular recording electrode, which could be prevented by using a zinc chelator (Trombley et al., 2011).

Several studies have utilized metal-chelators including tricine (Kirson et al., 2013; McCracken et al., 2013b; Cornelison et al., 2017) ethylenediaminetetraacetic acid (EDTA) (Kay, 2004; Trombley et al., 2011) and ZX1 (Perez-Rosello et al., 2015; Zhang et al., 2016) in attempts to remove contaminating zinc from recording solutions. However, these chelators are not completely selective and can disrupt functional studies by chelating other divalent ions (Ramos Silva et al., 2001; Radford and Lippard, 2013). While many studies suggest the chelators have minimal impact on baseline controls, it does not negate the possibility that chelators may interact with other modulators in solution, alter modulator binding or modulate the GlyR directly. Other groups instead utilize zinc-insensitive mutants, such as GlyRα_1_ W170S, to screen positive allosteric modulators (PAMs) (Cornelison et al., 2017).

As zinc is a ubiquitous contaminant in many buffers, purified products and common plastic labware (Kay, 2004; Cornelison and Mihic, 2014), it has previously been suggested to impact on the results of functional GlyR studies and may inadvertently cause the effectiveness of some GlyR modulators to be over-estimated. This could greatly impact the assessment of potential drug-candidates and contribute to the reduced effectiveness of compounds that reach clinical stages. This is especially true for GlyR modulators being developed for pain therapeutics due to the changes in spinal zinc concentrations that have been observed during chronic pain conditions (Jo et al., 2008). We have previously demonstrated that a series of *N*-acyl-amino acids potentiate glycine activation of GlyRs (Gallagher et al., 2020, 2023), but we have not established the extent to which contaminating zinc ions may influence the activity of these bioactive lipids. In this study, methods to negate the impact of contaminating zinc in functional studies of GlyRs are explored in order to characterize the positive allosteric modulation of GlyRs by *N*-acyl amino acids.

## 2 Methods

### 2.1 Materials

All chemicals including *N*-oleoyl glycine (NOGly) were obtained from Sigma (Sydney, Australia) unless otherwise stated. 2-[8-(2-octylphenyl)octanoylamino] acetic acid (8–8 OPGly) was synthesized as previously described (Gallagher et al., 2023). For all experiments zinc refers to the use of zinc chloride (ZnCl_2_). Zinc and metal chelators; tricine and Ca-EDTA were stored as stock solutions dissolved in frog Ringer’s solution (ND96: 96 mM NaCl, 2 mM KCl, 1 mM MgCl_2_, 1.8 mM CaCl_2_, 5 mM hemi-Na + -HEPES (Genscript, NJ, U.S.A.), pH 7.5) at 4°C. Stock solutions of both chelators were pH adjusted to 7.5, such that addition of tricine and Ca-EDTA would not alter the pH of recording solutions (Supplementary Table S2). Lipid modulators were stored at −20°C in DMSO and thawed once, on the day of use.

### 2.2 Synthesis of WT and mutant RNA

pGEMHE-h plasmids containing the cDNA of GlyR subunits α_1_b, and α_3_L (herein referred to as α_1_, and α_3_) were provided by Mary Collins (Faculty of Pharmacy, University of Sydney). Mutations were inserted into wild-type (WT) cDNAs through site directed mutagenesis, performed using oligonucleotide primers obtained from Sigma Aldrich (Sigma, Australia), the Q5 site-directed mutagenesis kit (New England Biolabs, Victoria, Australia) and MJ mini personal thermal cycler (Bio-rad, Hercules, CA, United States). Plasmids and PCR products were transformed into DH5α component *Escherichia coli* (*E. coli*) cells and DNA was extracted and purified using the Pure Link Quick Plasmid Purification kit (Life Technologies, Löhne, Germany). Sequences were confirmed by the Australian Genome Research Facility (Sydney, Australia).

For use in electrophysiology experiments, WT and mutant DNA were linearized using the NheI restriction enzyme (New England Biolabs, MA, USA) and RNA was transcribed with the T7 RNA polymerase mMessage mMachine Kit (Ambion, TX, USA). DNA and RNA concentrations were determined using a microvolume spectrophotometer (Thermo Scientific NanoDrop, Sydney, NSW, Australia) and stored at −20°C.

### 2.3 Two-electrode voltage clamp electrophysiology

*Xenopus laevis* (RRID:NCBITaxon_8355) were obtained from NASCO (Fort Atkinson, WI, U.S.A.). All surgical procedures were conducted as described previously (Vandenberg et al., 1998), in accordance with the Australian Code of Practice for the Care and Use of Animals for Scientific Use and approved by the University of Sydney Animal Ethics Committee. Briefly, female *Xenopus laevis* frogs were anesthetised using 0.17% 3-aminbenzoic acid ethyl ester (Tricaine) for 12 min. Small incisions (1–1.5 cm) were made in the skin and muscle layers, in the lateral portion of the lower abdomen and ovarian sacs were extracted using forceps. Ovarian sacs were disrupted through mechanical agitation with surgical scissors and individual oocytes were detached from follicular cells by enzymatic digestion with 3 mg/mL collagenase A (Boehringer, Mannheim, Germany) at 18°C for 45–90 min. Oocytes were then rinsed thoroughly with OR-2 solution (82.5 mM NaCl, 2 mM KCl, 1 mM MgCl•6H2O, 5 mM hemi-Na + -HEPES, pH 7.5) and subsequently rinsed in storage solution which consisted of ND96 supplemented with 50 μg/mL gentamycin, 2.5 mM sodium pyruvate, 100 μM/mL tetracyclin and 0.5 mM theophylline.

Defolliculated stage IV-V oocytes were microinjected with 4.6 ng of GlyR RNA in a 23 nL volume (Drummond Nanoject, Drummond Scientific Co., Broomall, PA, U.S.A.) and stored on a platform shaker (Ratek, Victoria, Australia) at 18°C in ND96 storage solution. Oocytes were rinsed daily with ND96 storage solution. GlyR expression levels were sufficient to measure receptor activity 2–6 days following RNA injection.

During recording, oocytes were continually perfused with ND96 solution within a 500 μL recording chamber. All recording was conducted within ND96 solution unless otherwise stated. Microelectrodes were fabricated from 1 mm borosilicate capillary tubes (Harvard Apparatus, Holliston, MA, United States) using a single-stage glass micropipette puller (Narasihge Japan, Tokyo, Japan) and filled with 3 M KCl. Whole cell currents were measured at −60 mV using a Geneclamp 500B amplifier (Axon Instruments, Foster City, CA, United States) interfaced with a Powerlab 2/26 chart recorder (ADI Instruments, Sydney, Australia) and analysed using LabChart software (ADI Instruments). All data was analysed using Graph Pad Prism 9.0 (GraphPad Software, San Diego, CA, United States).

### 2.4 Estimation of zinc concentrations in buffers

Buffers were diluted 1:10 with 2% HNO_3_ and analyzed on an Agilent 7,700 s ICP-MS (Mulgrave, Vic, Australia). Samples, standards and a 50 ng mL^−1^ Rhodium internal standard were carried via a three-channel peristatic pump and introduced into the ICP-MS via a MicroMist nebulizer and Scott type double pass spray chamber, cooled to 2°C. External calibration was performed using a 5-point (0.6–60 ppb) calibration curve. Agilent Technologies ICP-MS Chemstation software was used for all instrument control and data analysis. All acids and standards used were trace-metal grade and purchased from Choice Analytical (Thornleigh, NSW, Australia).

### 2.5 Recording methods and data analysis

For all recording methods, example traces can be found in the figures associated with the results. For all statistical tests throughout the paper, a *p*-value of <0.05 was taken to be statistically significant. Statistical significance is represented as ns or not shown for non-significant results, and * = *p* ≤ 0.05, ** = *p* ≤ 0.01, *** = *p* ≤ 0.001 and **** = *p* ≤ 0.0001 for significant result.

#### 2.5.1 Agonist concentration-responses

WT and mutant GlyR functionality were initially characterized by measuring agonist concentration-dependent currents in ND96. Increasing concentrations of glycine were applied to an oocyte until peak currents were achieved. Peak currents were measured with a 2-min wash-out period between low concentrations (<EC_10_) and a 5-min wash-out period between high concentrations (>EC_10_) to ensure full re-sensitisation of the receptors. Data was fitted using a modified Hill equation with a variable slope linear-regression model shown in Equation 1:


(1) $$\frac{I}{I_{\text{max}}}=\frac{{\left[\mathrm{Agonist}\right]}^{n}}{{\left[\mathrm{Agonist}\right]}^{n}+E{C_{50}}^{n}}$$

Where *I* represents the current (nA) and *I_max_* is the maximum current generated by the agonist glycine, *n* represents the Hill coefficient, [*Agonist*] indicates the concentration of glycine applied and *EC_50_* signifies the concentration of the agonist that produces 50% of the maximum response. Individual cells are normalized to their peak currents and data is expressed as mean ± SEM with an n ≥ 5 obtained from at least two independent batches of oocytes, unless otherwise stated. Parameters determined from curve fitting including peak currents, Hillslope values and agonist EC_50_ concentrations were compared between receptors using t-tests.

#### 2.5.2 Modulator concentration-responses

Concentration responses of modulators including zinc and metal chelators were measured using a cumulative application. Increasing concentrations of a modulator were co-applied to a steady current elicited by an EC_5_ concentration of glycine, with each concentration being applied until a plateau in response was achieved. The currents achieved at each concentration were then calculated as a percentage of the agonist current in the absence of modulator to provide the degree of potentiation. These values were then plotted using a modified Hill equation with a variable slope linear-regression model shown in Equation 2:


(2) $$\frac{I}{I_{\text{max}}}=\frac{{\left[\mathrm{PAM}\right]}^{n}}{{\left[\mathrm{PAM}\right]}^{n}+E{C_{50}}^{n}}$$

Where *I* represents the current in nA and *I_max_* is the maximum current generated by the agonist, *n* represents the Hill coefficient, [*PAM*] indicates the concentration of modulator applied and *EC*_50_ signifies the concentration of the agonist that produces 50% of the maximum degree of potentiation.

For biphasic concentration responses that could not be plotted using a using a modified Hill equation with a variable slope linear-regression model, data was plotted using a bell-shaped linear regression model utilizing Equations 3–7:


(3) $$\mathrm{Spa}n_{1}=\mathrm{Platea}u_{1}-\mathrm{Dip}$$

(4) $$\mathrm{Spa}n_{2}=\mathrm{Platea}u_{2}-\mathrm{Dip}$$

(5) $$\mathrm{Sectio}n_{1}=\frac{\mathrm{Spa}n_{1}}{1+{\left(\frac{E{C_{50}}_{\left(1\right)}}{X}\right)}^{nH_{1}}}$$

(6) $$\mathrm{Sectio}n_{2}=\frac{\mathrm{Spa}n_{2}}{1+{\left(\frac{X}{E{C_{50}}_{\left(2\right)}}\right)}^{nH_{2}}}$$

(7) $$Y=\mathrm{Dip}+\mathrm{Sectio}n_{1}+\mathrm{Sectio}n_{2}$$

Where *Plateau_1_* and *Plateau_2_* are the plateaus at the left and right ends of the curve in the same units as Y, *Dip* is the plateau in the center of the curve in the same units as Y, $E{C_{50}}_{\left(1\right)}$ and $E{C_{50}}_{\left(2\right)}$ are the concentrations that give the half-maximal stimulatory and inhibitory effects in the same units as X, and *nH_1_* and *nH_2_* are the hill coefficients for the stimulatory and inhibitory effects. Multiple t-tests were used to compare the modulator at each concentration tested, between the two receptor subtypes.

#### 2.5.3 Characterization of bioactive lipid PAMs

For this study, the bioactive lipid PAMs NOGly and 8–8 OPGly were screened for activity at a single 1 μM concentration. An approximate EC_5_ glycine concentration was initially applied until a stable glycine response was achieved. 1 μM concentration of the bioactive lipid PAM was then co-applied until a stable response was achieved. Following this, an approximate EC_5_ glycine concentration was re-applied in the absence of the bioactive lipid PAM to give an indication of its reversibility. The degree of modulation was calculated using Equation 8:


(8) $$\%\mathrm{Modulation}=\frac{I_{\mathrm{PAM}}-I_{gly}}{I_{gly}}\times 100$$

Where *I_PAM_* is the current (nA) generated by the co-application of glycine and the PAM, and *I_gly_* is the current (nA) generated by glycine alone. To compare the activity of the bioactive lipid PAM between receptor subtypes, all data points were normalized to the mean modulation incurred by the PAM on the WT receptor. Values were then compared between receptor subtypes using a t-test.

For bioactive lipid PAMs screened in the presence of metal chelators on the same cell, the method mentioned above was initially conducted. The cell was then perfused with ND96 for 5 min to allow re-sensitisation of the receptors. A set concentration of metal chelator in ND96 was then applied for an additional 5 min and the modulator-screening method was repeated in the presence of the metal chelator. The degree of modulation and the raw current values in the absence and presence of the modulator were then normalized to the values obtained on the WT receptor, and compared between receptor subtypes using t-tests.

#### 2.5.4 Zinc and bioactive lipid PAM synergism

The potential for synergistic interactions between zinc and the bioactive lipid PAM was assessed by comparing the modulatory activity of both modulators co-applied, to the sum of their individual modulatory activities. The individual activities of the PAMs were first assessed on the same cell using the aforementioned screening method. The sum of these two values is the estimate of modulatory activity. Both modulators were then co-applied utilizing the same screening method and the degree of potentiation was compared to the estimated potentiation using a paired t-test.

## 3 Results

### 3.1 Zinc concentration in solutions used in this study

It has been reported that zinc is a common contaminant of biological solutions and laboratory plasticware may be a source of contamination. We tested the stock solutions of glycine, tricine and Ca-EDTA as well as the oocyte storage solution. In addition we sampled the ND96 recording solution after it had passed through plastic tubing and entered the oocyte recording chamber. Zinc concentrations in stock solutions, as determined by ICP-MS, were as follows: Tricine = 336 nM; Ca-EDTA = 581 nM; Glycine = 433 nM. Oocyte storage solution = 4.58 μM; ND96 = 489 nM; These values are consistent with previously reported values for similar solutions (Kay, 2004; Cornelison and Mihic, 2014), and provide an indication of what may be expected in the recording solutions. While it is clear that zinc is present at low concentrations in the solutions used in this study, it is difficult to estimate the precise concentration that will be present under our recording conditions because of the likely low rate of washout of zinc when tightly bound to the receptor. Thus, in the following experiments we assume that zinc is present but have not made further adjustments to zinc concentration estimates.

### 3.2 Characterization of zinc-insensitive mutants in GlyRα_1_ and GlyRα_3_

Mutant GlyRs that are insensitive to zinc would make useful tools for investigating the actions of PAMs. Previous studies have explored mutations within the potentiating binding site and identified a H215A mutation within GlyRα_1_ that reduces zinc potentiation, and similar mutations of aspartate (D194A) and glutamate residues (E192A) also abolish zinc potentiation (Miller et al., 2005). As E192 is a conserved residue across all GlyR subunits, we explored the E192A mutation in GlyRα_1_ and GlyRα_3_. The E192A mutation in GlyRα_1_ produced functional receptors, however its sensitivity to zinc was not significantly different to WT GlyRα_1_ at any zinc concentration tested (Supplementary Figure S1). For GlyRα_3_, the E192A mutant had significantly reduced zinc potentiation for concentrations up to 30 μM but significantly increased zinc potentiation at 100 μM (Supplementary Figure S2). Thus, the E192A mutation does not abolish zinc potentiation in either receptor, making this mutation unsuitable for further study.

The hyperekplexic W170S mutation has also been shown to abolish zinc potentiation of GlyRα_1_ expressed in HEK239T cells (Zhou et al., 2013) and oocytes (Cornelison et al., 2017), and has been used in functional studies to test for interactions between zinc and PAMs of GlyR (Cornelison et al., 2017). The W170 residue is conserved across all GlyR subunits (Figure 1) however, its ability to disrupt zinc potentiation has only been assessed in GlyRα_1_. We therefore explored the W170S mutation in the α_1_ and α_3_ subunits. Unlike GlyRα_1_ W170S which produced robust concentration-dependent currents in response to glycine, GlyRα_3_ W170S did not produce any current in response to glycine up to 1 mM. This suggests the mutation greatly reduces the expression of the receptor or renders it non-functional. In GlyRα_1_, the W170S mutation did not significantly impact maximal currents but it significantly reduced glycine affinity, increasing the EC_50_ concentration from 17 μM to 48 μM (Supplementary Figure S3).

**Figure 2:**
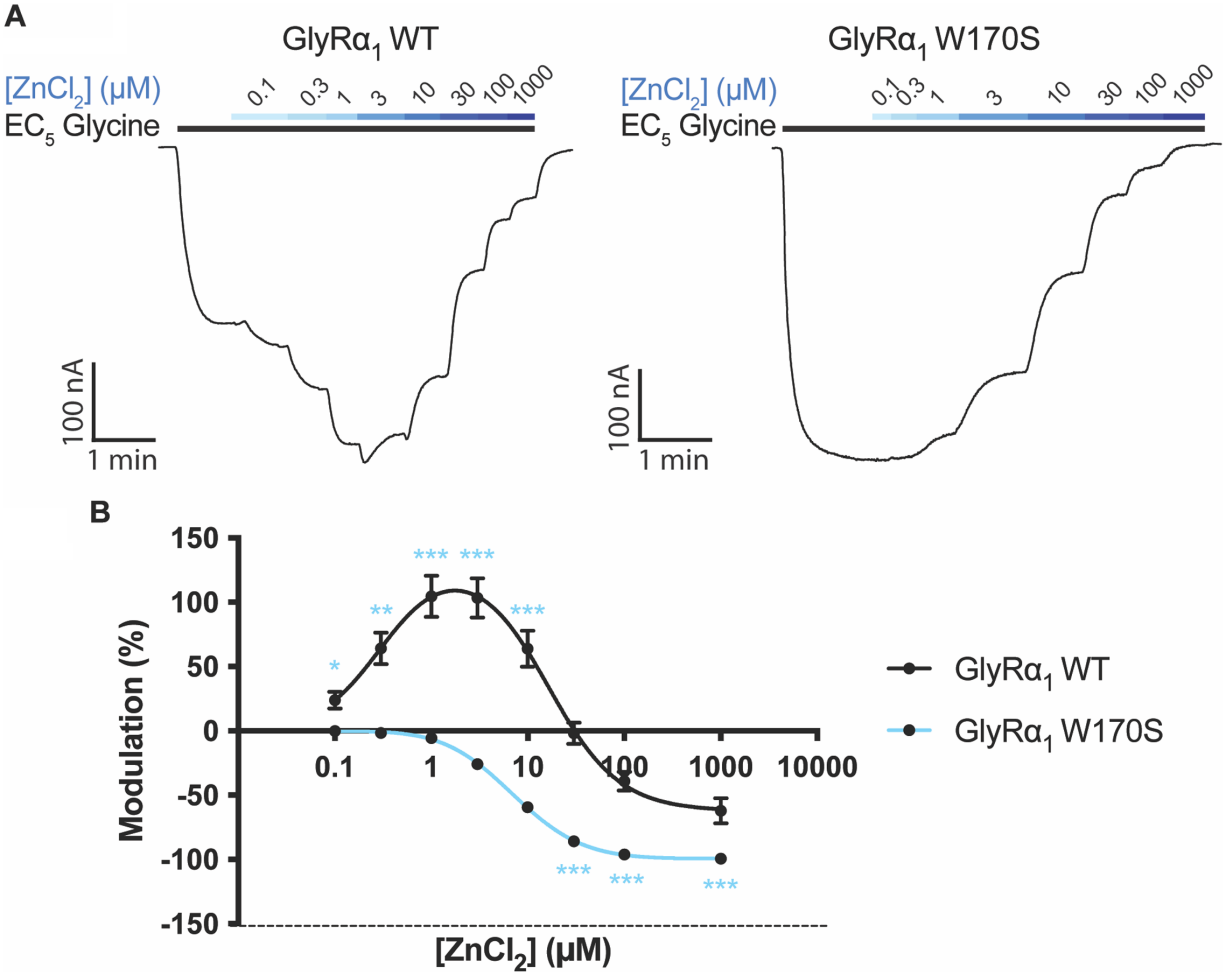
Zinc sensitivity of W170S mutation in GlyRα_1_. **(A)** Example traces of zinc concentration-responses conducted on GlyRα_1_ WT and GlyRα_1_ W170S. The EC_5_ concentration for GlyRα_1_ WT is 5 μM and GlyRα_1_ W170S is 12 μM. **(B)** Zinc concentration-responses are plotted for WT (black) and W170S (blue) GlyRα_1_. Data is plotted as mean ± SEM with an n ≥ 5 and fitted using a bell-shaped dose response model. The modulatory activity at each concentration is compared between the two receptors using multiple *t*-tests. The degree of significance is denoted as: * = *p* ≤ 0.05, ** = *p* ≤ 0.01, *** = *p* ≤ 0.001 and **** = *p* ≤ 0.0001.

The zinc sensitivity of GlyRα_1_ W170S was compared to WT GlyRα_1_ by measuring zinc concentration-responses in the presence of a low glycine concentration (EC_5_), with examples traces for WT and W170S shown in Figure 2. The W170S mutation in GlyRα_1_ abolished zinc potentiation and incurred significantly greater inhibitory activity, causing the zinc-induced modulation to be significantly different to GlyRα_1_ WT at every concentration tested (Figure 2). This is consistent with previous literature (Cornelison et al., 2017) and permits this receptor to be utilized as a ‘zinc-insensitive’ GlyR with respect to contaminating zinc concentrations, which are likely to be in the nanomolar range.

### 3.3 Characterizing the impact of zinc chelators on GlyRs

**Figure 3:**
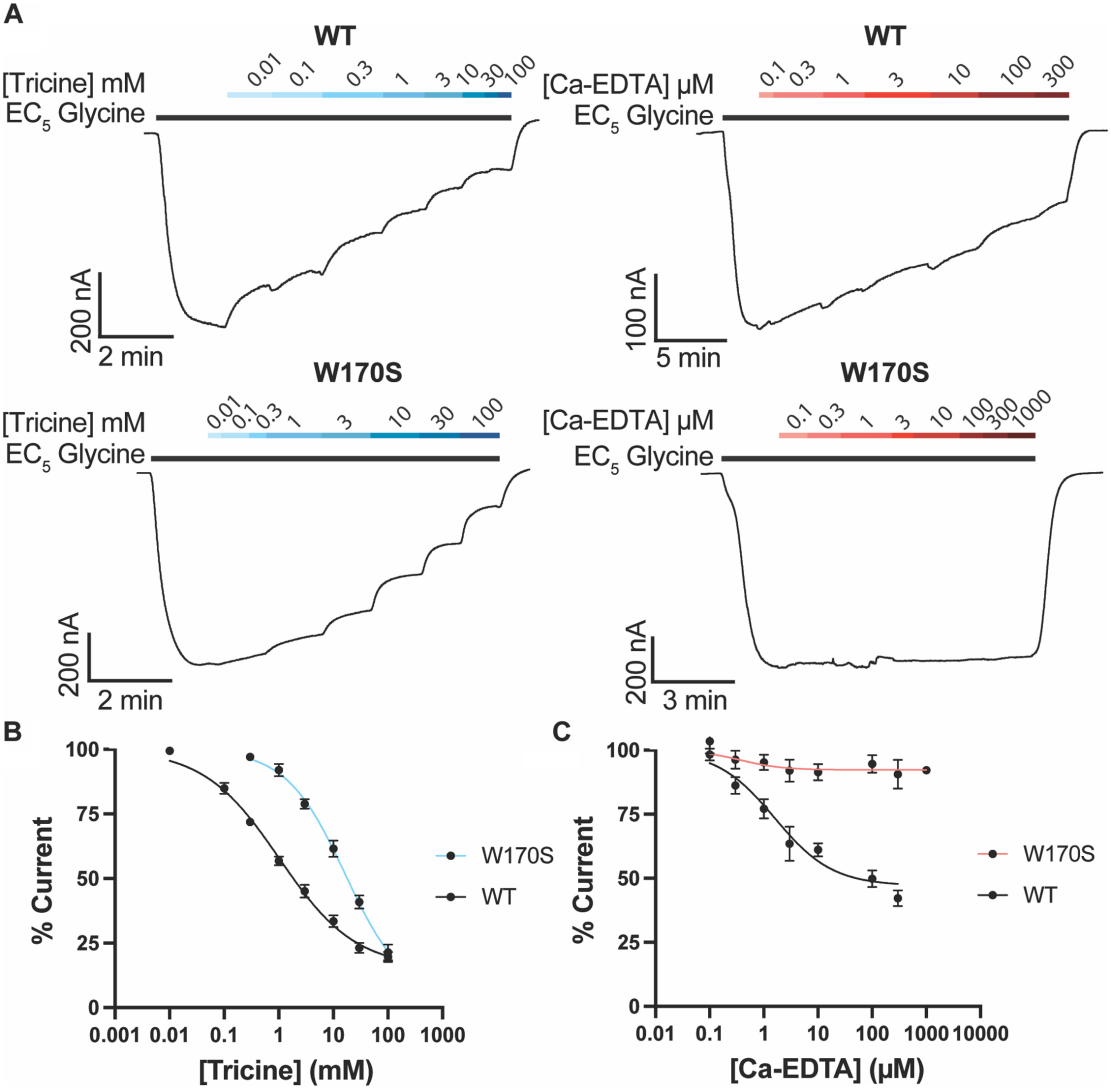
Impact of zinc chelators on GlyRα_1_. **(A)** Example traces illustrating the co-application of tricine (blue bars) and Ca-EDTA (red bars) on a current elicited by an EC_5_ concentration of glycine (black bar) for WT (5 μM) and W170S GlyRα_1_ (12 μM). Increasing concentrations of **(A)** tricine or **(B)** Ca-EDTA were co-applied with an EC_5_ concentration of glycine on WT and W170S GlyRα_1_. Data is normalized to the EC_5_ concentration of glycine for each cell. Data is plotted as mean ± SEM with an n ≥ 5 and fitted using a modified Hill equation with a variable slope linear-regression model.

Metal chelators are commonly added to buffers to remove metal ions such as zinc. A common chelator used for functional GlyR studies is tricine (Kirson et al., 2013; McCracken et al., 2013b; Cornelison et al., 2017). To determine the impact of tricine on GlyRα_1_, increasing concentrations of tricine were applied in the presence of a low glycine concentration (EC_5_) (Figure 3).

Tricine was found to inhibit glycine induced currents by up to 84.6 ± 2.8% with an IC_50_ of 1.0 ± 0.2 mM (Figure 3). To determine if this inhibition is due to zinc chelation or non-zinc mediated effects, the same experiment was also conducted on GlyRα_1_ W170S. Tricine was found to inhibit glycine induced currents on GlyRα_1_ W170S by up to 96.7 ± 10.4% with an IC_50_ of 16.6 ± 5.5 mM (Figure 3), indicating that there is a non-zinc mediated component to tricine inhibition at GlyRs.

Another chelator used to sequester divalent cations is EDTA. However, it is a non-specific chelator and can sequester other divalent cations in solution which may induce artifacts. Ca^2+^ in particular is present in recording solutions such as ND96 and can impact the stability of oocyte membranes (Wozniak et al., 2017). To minimize the sequestering of Ca^2+^, a pre-loaded form of EDTA, Ca-EDTA, can be used. Ca-EDTA inhibited glycine induced currents on WT GlyRα_1_ by 52.9 ± 4.6% with an IC_50_ of 1.6 ± 0.6 μM (Figure 3), however the inhibitory effects of Ca-EDTA were abolished in GlyRα_1_ W170S (max inhibition of 7.3 ± 2.1%) (Figure 3). This suggests that the inhibitory effects of Ca-EDTA on WT GlyRα_1_ are due to chelation of zinc ions and will be a more useful chelator than tricine when looking at other modulators of GlyRs.

### 3.4 Stimulation of GlyRs by NOGly and 8–8 OPGly in the presence and absence of zinc

**Figure 4:**
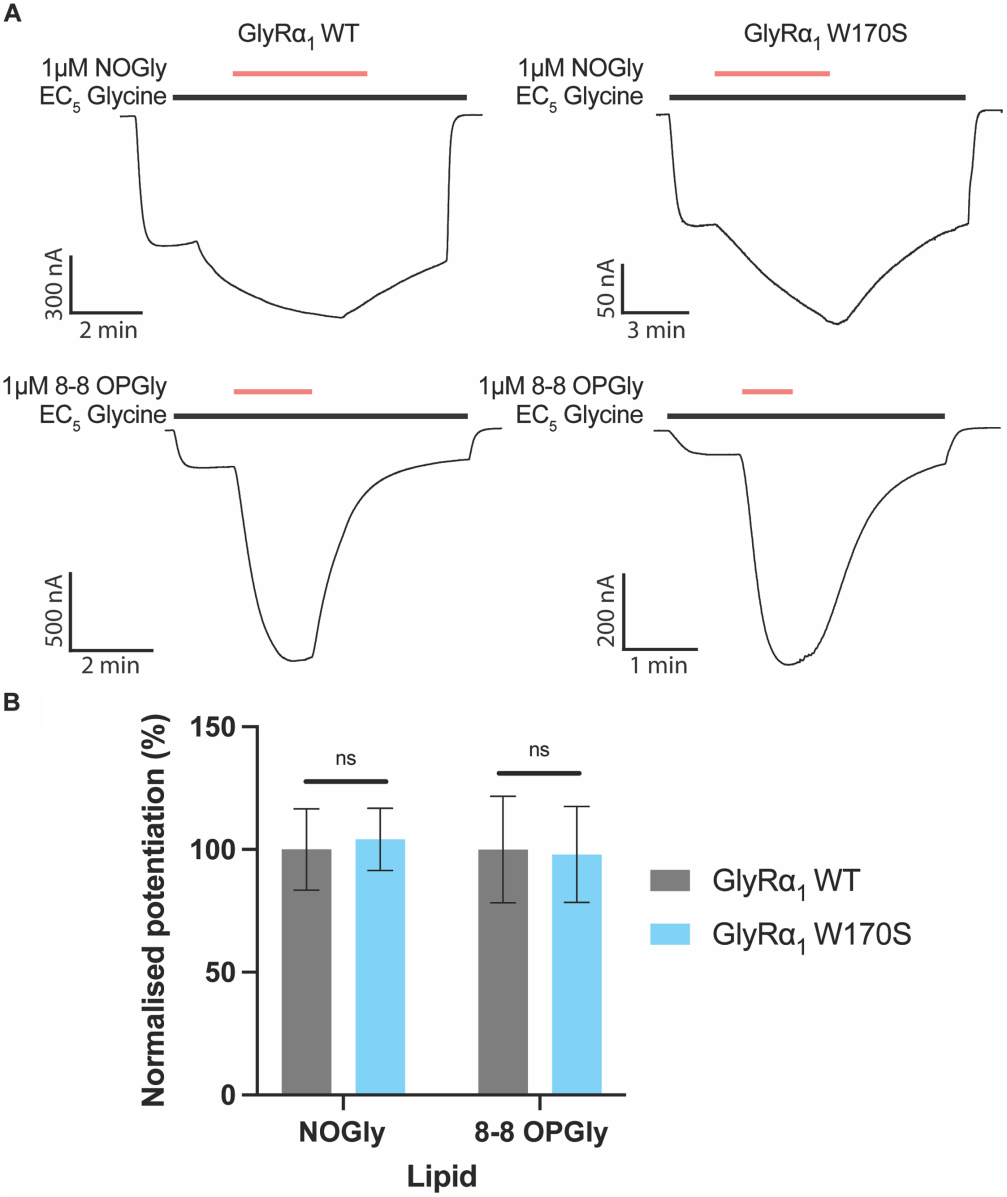
Impact of the zinc insensitive W170S mutation on lipid modulation of GlyRα_1_. C18 ω9 Glycine is represented as NOGly and 8–8 *ortho* phenylene glycine is represented as 8–8 OPGly. **(A)** Example traces illustrating the co-application of 1 μM NOGly or 1 μM 8–8 OPGly (red bars) on currents elicited by an EC_5_ glycine concentration (black bar) on WT (5 μM) and W170S GlyRα_1_ (12 μM). **(B)** Degree of potentiation induced by 1 μM NOGly and 1 μM 8–8 OPGly on WT (Gray) and W170S (Blue) GlyRα_1_. Data is normalized to the degree of potentiation on WT GlyRα_1_ and is compared using a t-test. Data is represented as mean ± SEM with an n ≥ 5. The degree of significance is denoted as: * = *p* ≤ 0.05, ** = *p* ≤ 0.01, *** = *p* ≤ 0.001 and **** = *p* ≤ 0.0001.

Zinc has previously been shown to impact the activity of PAMs and may synergistically enhance their modulatory effects (McCracken et al., 2013a,b). The activity of PAMs which have been assessed in experimental set-ups that have contaminating zinc could therefore be inadvertently over-estimated. Our lab has recently published studies on a series of acyl- and phenylene-containing bioactive lipids which act as PAMs at GlyRs (Gallagher et al., 2020, 2023). To determine if the activity of these PAMs were impacted by contaminating zinc, the activity of the most efficacious modulator 8–8 OPGly and NOGly were compared between WT GlyRα_1_ and the W170S mutant. The degree of potentiation induced by 8–8 OPGly or NOGly at GlyRα_1_ W170S were not significantly different from the potentiation induced on the WT receptor (Figure 4), which suggests that the W170S mutation does not influence the actions of 8–8 OPGly or NOGly.

**Figure 5:**
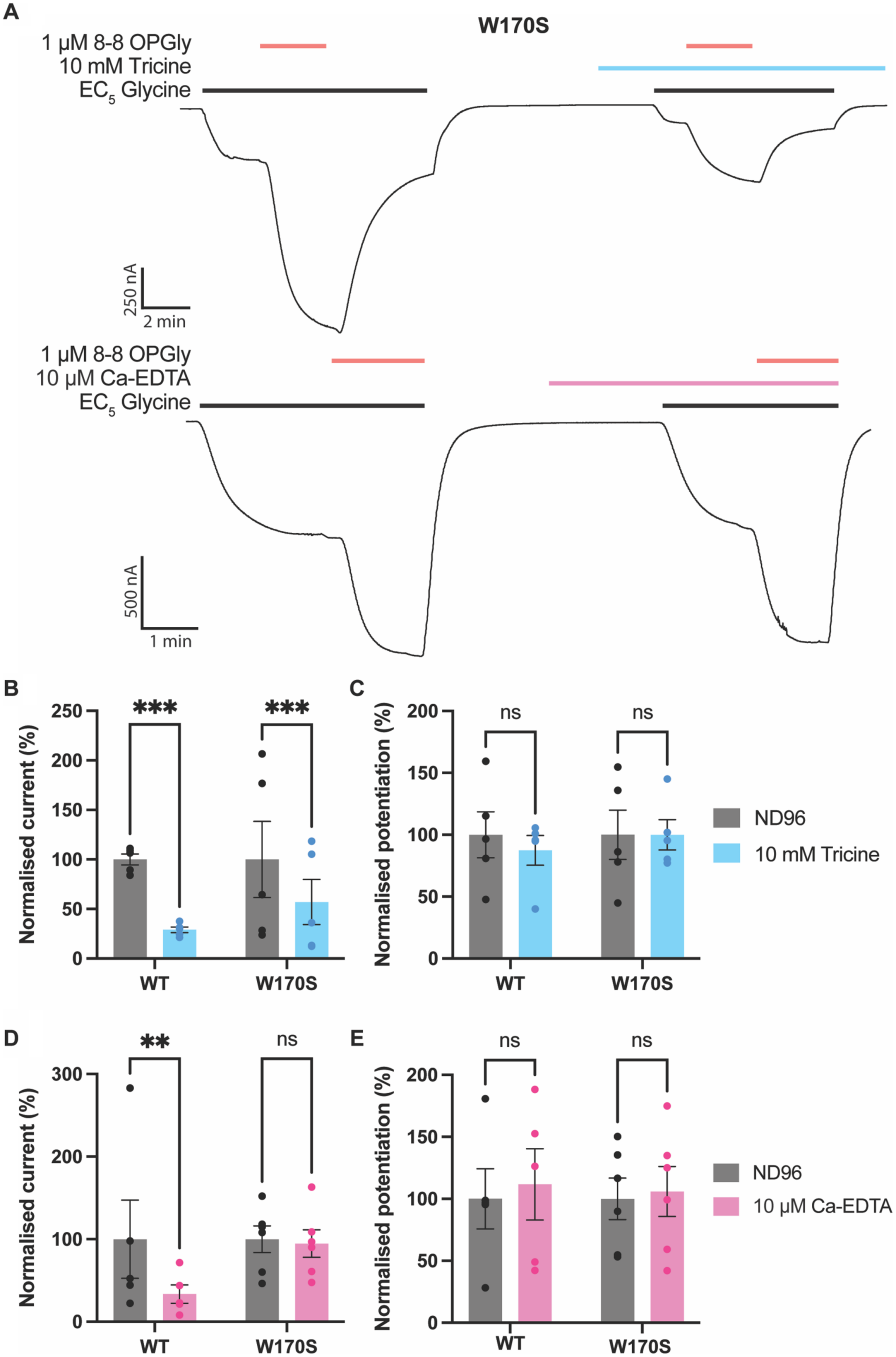
Impact of zinc chelators on lipid modulation of GlyRα_1_. 8–8 *ortho* phenylene glycine is represented as 8–8 OPGly. **(A)** Example traces illustrating the co-application of 8–8 OPGly (red bars) with an EC_5_ concentration of glycine (black bars) in the absence and presence of tricine (blue bar) or Ca-EDTA (pink bar) for GlyRα_1_ W170S. Raw current elicited by an EC_5_ concentration of glycine in the absence (ND96:Gray) and presence of **(B)** 10 mM Tricine (Blue) or **(D)** 10 μM Ca-EDTA (Pink) conducted on the same cell. The EC_5_ concentration for GlyRα_1_ WT is 5 μM and GlyRα1 W170S is 12 μM. Data is normalized to the current in the absence of chelator and is compared using a paired *t*-test. Potentiation induced by a 1 μM concentration of 8–8 OPGly in the absence (ND96:Gray) and presence of **(C)** 10 mM Tricine (Blue) or **(E)** 10 μM Ca-EDTA (Pink) conducted on the same cell. Data is normalized to the lipid potentiation in the absence of chelator and is compared using a paired t-test. For all panels data is represented as mean ± SEM with an n ≥ 5. The degree of significance for statistical tests is denoted as: ns = not significant, * = *p* ≤ 0.05, ** = *p* ≤ 0.01, *** = *p* ≤ 0.001 and **** = *p* ≤ 0.0001.

To further examine the impact of zinc and zinc-chelators on lipid-mediated potentiation of GlyRs, the potentiating effects of 8–8 OPGly were assessed in the absence and presence of metal chelators on the same cell expressing the W170S mutant (Figure 5). 1 mM tricine significantly reduced the raw currents induced by the EC_5_ concentration of glycine by over 50% however, when 8–8 OPGly was co-applied the degree of potentiation was very similar for both receptors. In comparison, 10 μM Ca-EDTA, which causes ~45% inhibition of WT GlyRα_1_, had no effect on the W170S mutant and the level of 8–8 OPGly stimulation also remained the same. Thus, we can conclude that the stimulatory effects of 8–8 OPGly are independent of the actions of any contaminating zinc.

**Figure 6:**
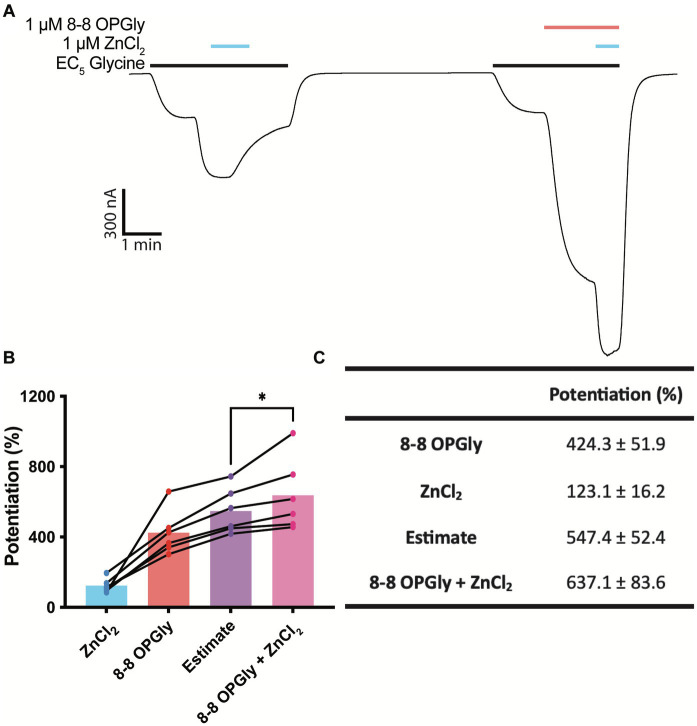
Synergistic interaction between zinc and 8–8 *ortho* phenylene glycine at GlyRα_1_. 8–8 *ortho* phenylene glycine is represented as 8–8 OPGly. **(A)** Example trace illustrating the individual application of 1 μM zinc (blue bar) and 1 μM 8–8 OPGly (red bar) on a current elicited by an EC_5_ concentration of glycine (black bar, 5 μM), and the co-application of both modulators. **(B)** Degree of potentiation induced by 1 μM zinc (blue), 1 μM 8–8 OPGly (red) or 1 μM of both modulators co-applied (pink) on the same cell. The sum of the two modulators applied individually is shown as the estimate (purple). The degree of potentiation in the co-application condition and the estimate are compared using a paired *t*-test. The degree of significance is denoted as: * = *p* ≤ 0.05, ** = *p* ≤ 0.01, *** = *p* ≤ 0.001 and **** = *p* ≤ 0.0001. **(C)** Potentiation induced in each condition. Data is represented as mean ± SEM with an n ≥ 5.

Zinc synergistically enhances the positive allosteric effects of ethanol and anesthetics on GlyRs (Cornelison et al., 2017) and while the basal, or contaminating, concentration of zinc in our recording system does not impact 8–8 OPGly activity, we wished to investigate whether higher concentrations of zinc will influence 8–8 OPGly activity. 1 μM zinc was used to test for possible synergistic interactions as synaptic zinc concentrations have been shown to rise to at least 1 μM following synaptic simulation, when conducted in an artificial synapse (Zhang et al., 2016). Both modulators (zinc, 8–8 OPGly) were applied independently, and the sum of their individual activities were used to calculate an estimate of the modulatory activity that would be expected if there were no synergism between the two. Both modulators were then co-applied, and the degree of potentiation achieved by their co-application was compared to the sum of the individual applications. 1 μM Zinc significantly enhances the potentiation induced by 1 μM 8–8 OPGly at GlyRα_1_ when analysed using a paired t-test (Figure 6). The degree potentiation elicited by the co-applications of zinc and 8–8 OPGly was 90% greater than the sum of the individual components. This ranged from 24 - 246% for the 6 cells.

## 4 Discussion

Zinc is a ubiquitous metal cation found throughout nature that is able to interact with a range of proteins, including GlyRs. In a laboratory setting, contaminating zinc concentrations are estimated to reach the nanomolar range (Cornelison and Mihic, 2014) which is sufficient to potentiate GlyRs and may influence the activity of GlyR modulators. It is important to address the effect zinc ions may have on the functional properties of GlyRs and how this may impact on GlyR-targeted drug-development.

### 4.1 Zinc chelators and zinc insensitive mutants

Zinc chelators have previously been used to remove contaminating zinc ions from recording solutions. One of the most commonly used chelators is tricine (Kirson et al., 2013; McCracken et al., 2013a; Cornelison et al., 2017), which is a derived from tris and glycine and was originally synthesized as one of Good’s buffers (Good et al., 1966). In this study we have demonstrated that tricine significantly reduces the glycine-induced currents of GlyRα_1_ by over 80%. This could be due the chelation of contaminating zinc ions present within the ND96 solution which would produce inhibition by removing zinc-mediated potentiation of the receptor. To explore this, the effect of tricine was also assessed using the W170S GlyRα_1_, which abolishes zinc potentiation. The mutant receptor was similarly inhibited by tricine which demonstrates that tricine reduces receptor activity via a non-zinc mediated mechanism.

As tricine is an analog of glycine, its structural similarity may allow it to access and obstruct the agonist binding cavity. The tris-moiety is significantly larger than the amine group of glycine and if bound within the orthosteric binding cavity, would prevent the closure of loop-C which is required for receptor activation (Kumar et al., 2020; Yu et al., 2021). Tricine could therefore inhibit receptor activity by acting as a competitive antagonist. Tricine has also been shown to form complexes with glycine (Zayed and Ammar, 2014) which would reduce the concentration of free glycine in recording solutions and would reduce receptor activation. Additional studies which explore these possible inhibitory mechanisms are required to understand the impact tricine has on the GlyR.

It is also possible that tricine impacts GlyR activity through non-selective chelation of divalent cations. Tricine can also bind strongly to magnesium, calcium, cobalt, copper and nickel (Ferreira et al., 2015). While cobalt, copper and nickel are not commonly used in biological buffers or significantly impact GlyR activity, the recording solution ND96 contains 1 mM magnesium and 1.8 mM calcium. Chelation of these ions would alter the ionic composition of the buffer which could significantly impact on the function of the receptor. Previous studies have used other chelating agents such as EDTA (Kay, 2004; Trombley et al., 2011) and ZX1 (Perez-Rosello et al., 2015; Zhang et al., 2016), however they too have varying levels of selectivity. As a way to address this, we also explored the activity of Ca-EDTA which is pre-loaded with calcium and would not be expected to sequester calcium ions from ND96. 10 μM Ca-EDTA was found to inhibit the WT GlyRα1 by over 50%, but when applied to W170S GlyRα_1_ the inhibitory activity was abolished. This suggests the inhibitory effects of Ca-EDTA on the WT receptor are solely due to the chelation of contaminating zinc ions. The inhibition of the WT receptor also confirms the presence of contaminating zinc ions in ND96 solution which are causing a basal level of potentiation during recording.

An alternative approach to negate zinc contamination is the use of zinc-insensitive mutations. The W170S mutation within GLRA1 produces a hyperekplexic phenotype by removing zinc potentiation (Al-Futaisi et al., 2012; Zhou et al., 2013). W170 does not form part of the zinc binding site, but instead occurs on the β8-strand and sits parallel in between the β1 and β9-strands. The aromaticity of W170 likely influences the positioning of the β9-strand which binds zinc, and therefore indirectly contributes to the conformation of the zinc binding site responsible for potentiation. Within this study, the W170S mutation was found to completely abolish zinc potentiation in GlyRα_1_. This is consistent with previous literature (Cornelison et al., 2017) and allows this receptor to be used as a tool to assess zinc-contamination. However, it is important to note the mutation was found to reduce the affinity of glycine compared to the WT receptor. This could suggest the mutation impacts receptor functionality or may be a result of removing basal zinc potentiation caused by contaminating zinc, similar to the addition of Ca-EDTA.

### 4.2 Zinc and GlyR PAMs

Overall, these results indicate that the W170S mutation and the chelator Ca-EDTA are suitable methods for removing the impact of zinc contamination, and that tricine is not a suitable chelator for functional studies of GlyRα_1_. To further explore the utility of these methods in practice, all three methods were used to assess the impact of bioactive lipid PAMs of GlyRs. When the bioactive lipid PAMs NOGly and 8–8 OPGly were tested on W170S GlyRα_1_ they induced similar levels of potentiation as WT GlyRα_1_. This suggests that lipid modulation of GlyRs is independent from zinc modulation and does not require zinc to illicit potentiation. This is important for modulators which are being developed as possible pain therapeutics. 8–8 OPGly was also found to illicit similar levels of potentiation at GlyRα_1_ in the absence and presence of both chelators, despite the reduction in glycinergic current from zinc-chelation and tricine-induced inhibition. This further supports that lipid modulation of GlyRs is not impacted by contaminating zinc or by the inhibitory mechanism induced by tricine. Furthermore, this suggests that the degree of lipid modulation is not influenced by nanomolar concentrations of zinc which are likely to occur in biological solutions, and has not impacted the results of previous studies (Gallagher et al., 2020, 2023).

It is not only crucial to determine the activity of PAMs in the absence of zinc, but also beneficial to test novel PAMs in the presence of physiologically relevant zinc concentrations to mimic endogenous conditions. Endogenous zinc concentrations have previously been shown to synergistically enhance the activity of some PAMs. One example of this is ethanol, which was found to act synergistically with zinc at GlyRα1 when expressed in oocytes (McCracken et al., 2010). Additionally, when ethanol and volatile an aesthetics such as isoflurane, chloroform and halothane are tested on W170S GlyRα1, their potentiating activity is reduced, which does not occur for intravenous anesthetics such as toluene or trichloroethane (Cornelison et al., 2017). Developing modulators which interact synergistically with zinc could enhance their activity *in vivo*. Within this study a 1 μM concentration of zinc was used to test for possible synergistic interactions as synaptic zinc concentrations have been shown to rise to at least 1 μM following a single synaptic simulation, when conducted in an artificial synapse (Zhang et al., 2016). Similar to ethanol, the co-application of zinc and 8–8 OPGly was greater than the sum of their individual effects, which suggests a synergistic interaction is occurring (McCracken et al., 2010). It is unclear how zinc binding within the ECD mechanistically enhances the activity of lipid modulators which are expected to bind within the TM domains. Zinc binding may induce conformational changes that are transduced down into the TM domains via intermediate loops, which could enhance the efficacy of the lipid binding cavity. Zinc has also been suggested to act at an intracellular binding site within the ICD (Trombley et al., 2011) which could be partially responsible for the synergistic interaction. Regardless of its mechanisms, the synergistic interaction between the two modulators may be advantageous for analgesic drug development, as it suggests the endogenous activity of bioactive lipid PAMs will be enhanced *in vivo* at nociceptive synapses which contain zinc (Birinyi et al., 2001).

## 5 Conclusion

Previously, zinc chelators such as tricine have been used to navigate zinc modulation in functional studies. However, in this study tricine was found to significantly reduce GlyR activity through a non-zinc mediated mechanism. The use of well characterized zinc-insensitive mutations, such as the W170S mutation, in GlyRα_1_ was found to be a better method to remove the impact of zinc modulation on GlyRs. However, further studies are required to identify appropriate mutations for other GlyRs, especially GlyRα_3_. It is also crucial to validate these mutations across different expression systems. These mutations can also be used to assess the activity of zinc chelators and validate their use. This method was used to validate the chelator Ca-EDTA for GlyRα_1_. This allows for a dual approach utilizing both zinc-insensitive mutations and effective chelators in assessing the impact on zinc on novel PAMs.

These methods were used to assess the impact of zinc contamination on bioactive lipid PAMs and confirmed that their assessment has not been previously impacted by contaminating zinc modulation. Similar methodologies should also be utilized when assessing the activity of novel PAMs at GlyRs. Furthermore, when 8–8 OPGly was co-applied with a physiologically relevant concentration of zinc that occur at nociceptive synapses, the degree of potentiation was enhanced. This is suggestive of a synergistic interaction between zinc and lipid modulators which could enhance lipid modulation *in vivo* and may be beneficial for producing analgesic activity.

## Data availability statement

The original contributions presented in the study are included in the article/Supplementary material, further inquiries can be directed to the corresponding author.

## Ethics statement

The animal study was approved by University of Sydney Animal Ethics Committee. The study was conducted in accordance with the local legislation and institutional requirements.

## Author contributions

CG: Conceptualization, Data curation, Formal analysis, Investigation, Methodology, Validation, Writing – original draft, Writing – review & editing. TR: Conceptualization, Funding acquisition, Methodology, Resources, Supervision, Writing – review & editing. RV: Conceptualization, Data curation, Formal analysis, Funding acquisition, Investigation, Project administration, Resources, Supervision, Writing – original draft, Writing – review & editing. DB: Data curation, Formal analysis, Investigation, Methodology, Validation, Writing – review & editing. TL: Data curation, Formal analysis, Investigation, Methodology, Validation, Writing – review & editing.
