# Supplementary material for: Methods for negating the impact of zinc contamination to allow characterization of positive allosteric modulators of glycine receptors
Source: Front Mol Neurosci. 2024 Jun 24;17:1392715. doi: 10.3389/fnmol.2024.1392715 (PMC11228362; doi:10.3389/fnmol.2024.1392715)
Supplement: Supplementary file 1 [file Data_Sheet_1.pdf]

Supplementary Material

Methods for negating the impact of zinc contamination to allow characterization of positive allosteric modulators of glycine receptors

Casey I. Gallagher[1], David P. Bishop[2], Thomas E. Lockwood[2], Tristan Rawling[3], Robert J. Vandenberg[1*]

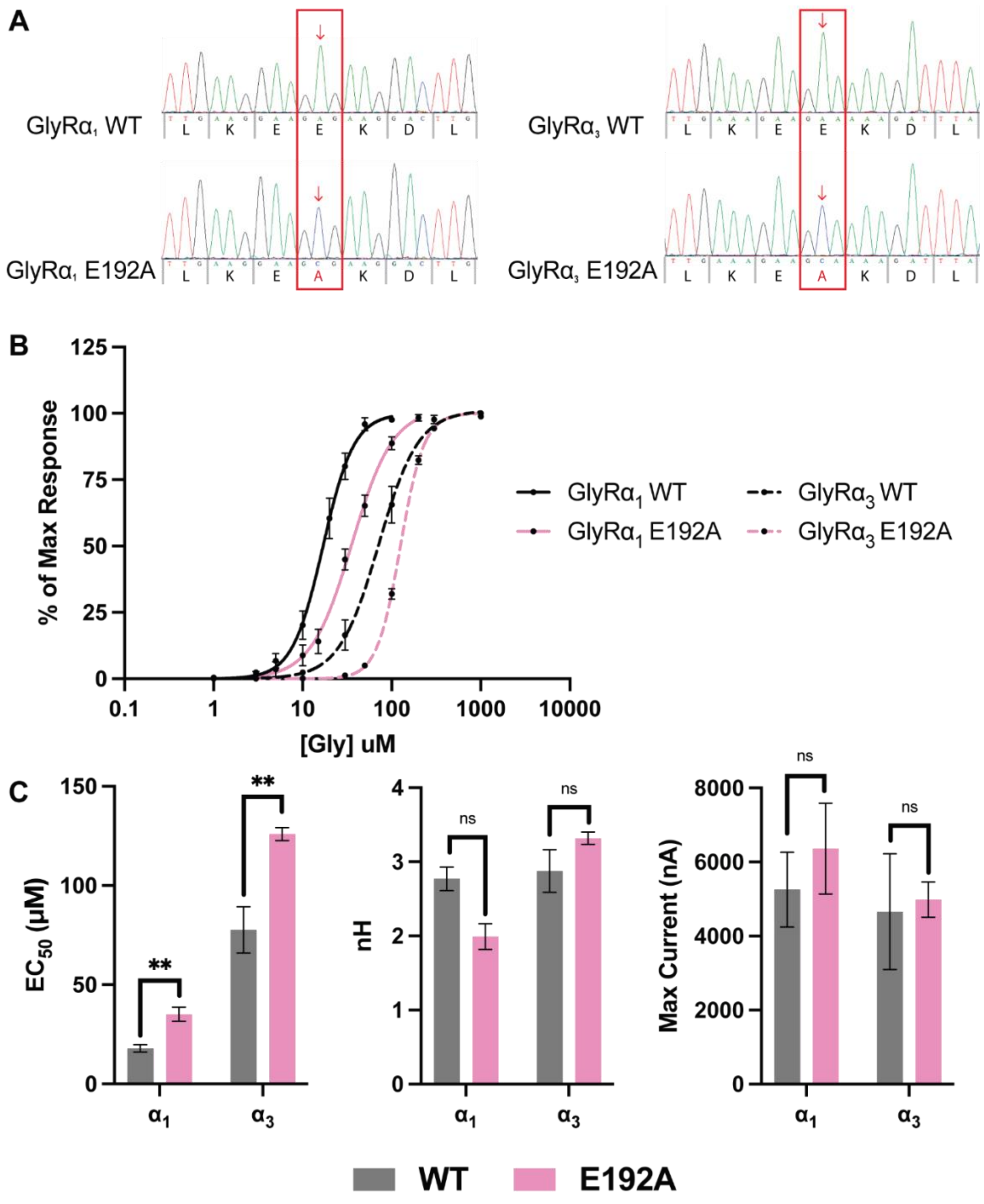

Figure S1. Characterisation of E192A mutation in GlyRα1 and GlyRα3. (A) Confirmation of inserted E192A mutation into GlyRα1 and GlyRα3 sequences. Red arrows indicate nucleotide differences between mutant and WT DNA. **(B)** Glycine concentration-responses are plotted for WT (black) and E192A GlyRs (pink). Data is plotted as mean ± SEM with an $n \geq 5$ and fitted using a modified Hill equation with a variable slope linear-regression model. **(C)** Comparison of EC_{50} concentrations, Hill coefficient values (nH) and maximum currents generated from linear-regression model for WT (grey) and E192A (pink) GlyRs. Data is plotted as mean ± SEM with an $n \geq 5$ and is

analysed using multiple t-tests. The degree of significance is denoted as: ns for not significant, * = $p \leq 0.05$, ** = $p \leq 0.01$, *** = $p \leq 0.001$ and **** = $p \leq 0.0001$.

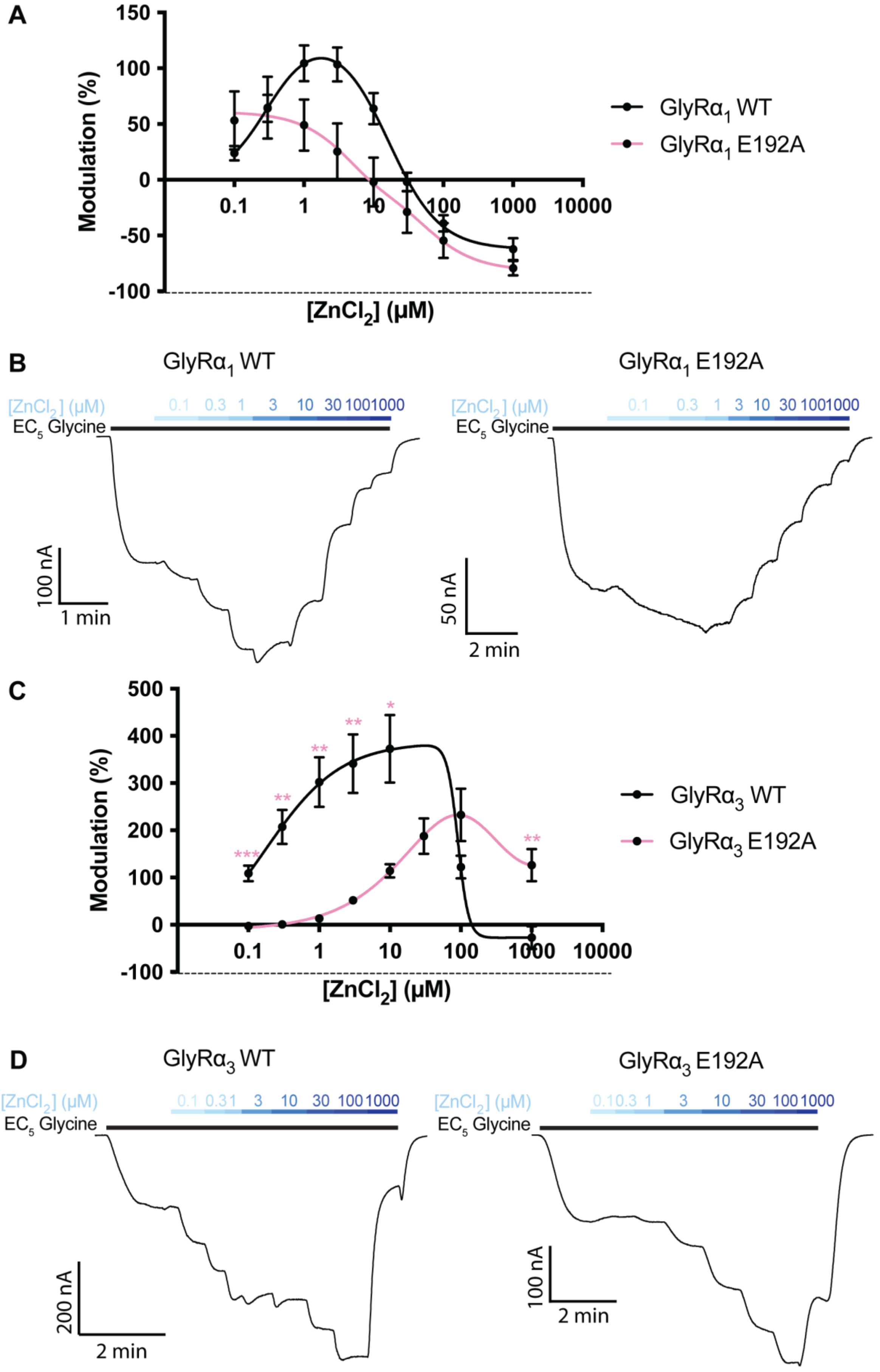
A
Modulation (%)
150
100
50
0
-50
-100
0.1
1
10
100
1000
10000
[ZnCl2] (μM)
GlyRα1 WT
GlyRα1 E192A
B
GlyRα1 WT
GlyRα1 E192A
[ZnCl2] (μM)
0.1 0.3 1 3 10 30 1001000
EC5 Glycine
100 nA
1 min
50 nA
2 min
C
Modulation (%)
500
400
300
200
100
0
-100
***
**
**
**
*
**
0.1
1
10
100
1000
10000
[ZnCl2] (μM)
GlyRα3 WT
GlyRα3 E192A
D
GlyRα3 WT
GlyRα3 E192A
[ZnCl2] (μM)
0.1 0.31 3 10 30 100 1000
0.10.3 1 3 10 30 100 1000
EC5 Glycine
200 nA
2 min
100 nA
2 min

Figure S2. Zinc sensitivity of E192A mutation in GlyRα$_1$ and GlyRα$_3$. Impact of the E192A mutation on zinc sensitivity in **(A)** GlyRα$_1$ and **(C)** GlyRα$_3$. The EC_5 glycine concentration for GlyRα$_1$ E192A is 8 µM and GlyRα$_3$ E192A is 50 µM. Zinc concentration-responses are plotted for WT (black) and E192A GlyRs (pink). Data is plotted as mean ± SEM with an $n \geq 5$ and fitted using a bell-shapes dose response model. The modulatory activity at each concentration is compared to WT using multiple t-tests. The degree of significance is denoted as: * = $p \leq 0.05$, ** = $p \leq 0.01$, *** = $p \leq 0.001$ and **** = $p \leq 0.0001$. Example traces of zinc concentration response conducted on WT and E192A (**B)** GlyRα$_1$ and **(D)** GlyRα$_3$.

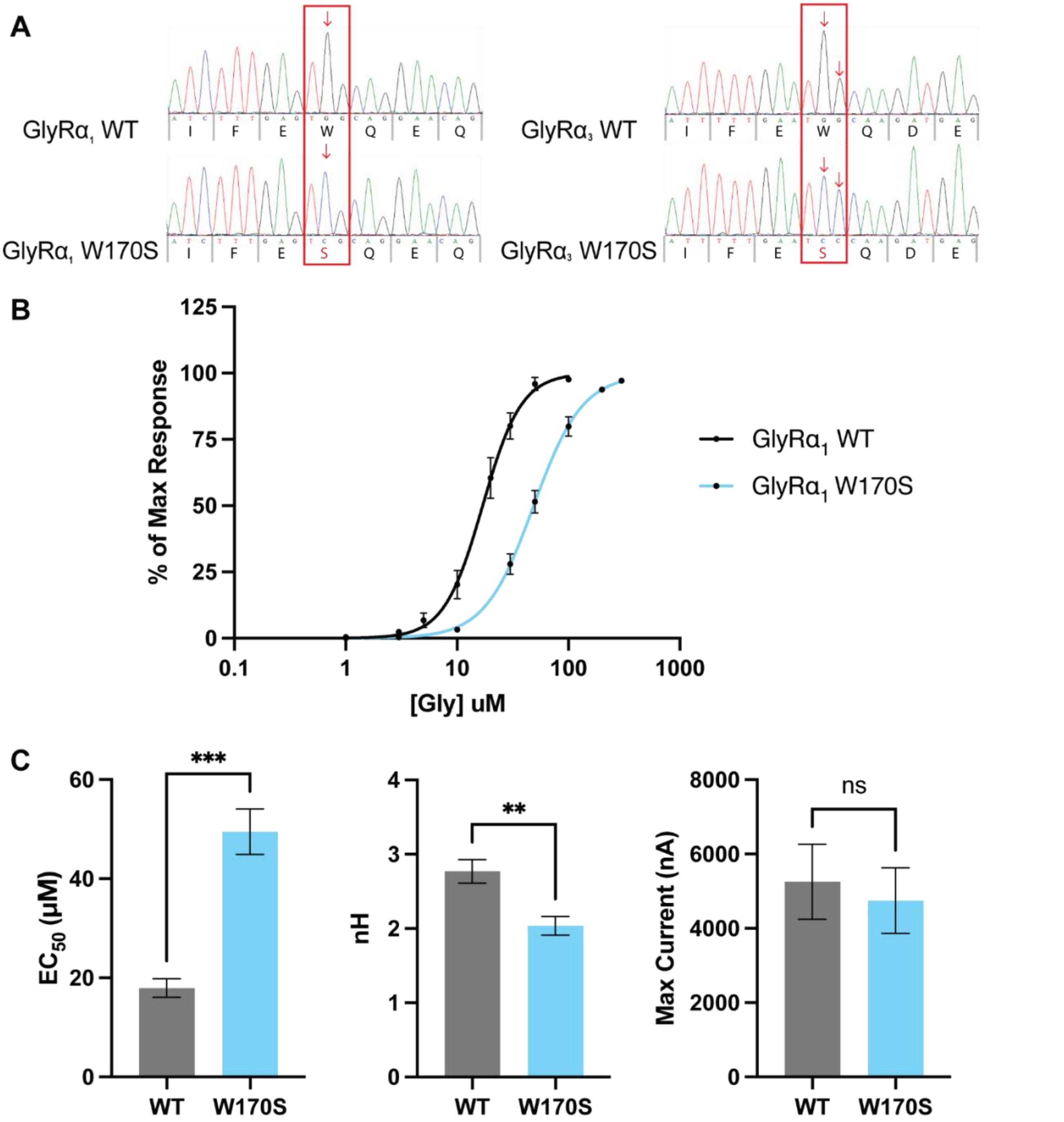

Figure S3. Characterisation of W170S mutation in GlyRα_1 and GlyRα_3. (A) Confirmation of inserted W170S mutation into GlyRα_1 and GlyRα_3 sequences. Red arrows indicate nucleotide differences between mutant and WT DNA. **(B)** Glycine concentration-responses are plotted for WT (black) and W170A GlyRα_1 (pink). Data is plotted as mean ± SEM with an $n \geq 5$ and fitted using a modified Hill equation with a variable slope linear-regression model. **(C)** Comparison of EC_{50} concentrations, Hill coefficient values (nH) and maximum currents generated from linear-regression model for WT (black) and W170A GlyRα_1 (pink). Data is plotted as mean ± SEM with an $n \geq 5$ and is analysed using a t-tests. The degree of significance is denoted as: ns for not significant, * = $p \leq 0.05$, ** = $p \leq 0.01$, *** = $p \leq 0.001$ and **** = $p \leq 0.0001$.

Table S1. Characterisation of WT and mutant GlyRs

		WT	W170S	E192A
α_1	Approx. EC_5 (µM)	5	12	8
	EC_{50} (µM)	17.94 ± 1.89	49.50 ± 4.56	35.11 ± 3.51
	nH	2.77 ± 0.16	2.04 ± 0.13	2.88 ± 0.29
	Max current (nA)	5253.63 ± 1009.10	4746.33 ± 882.92	4658.65 ± 1562.60
α_3	Approx. EC_5 (µM)	18	Non-expressing	50
	EC_{50} (µM)	77.63 ± 1.89		125.96 ± 3.24
	nH	2.68 ± 0.29		3.32 ± 0.08
	Max current (nA)	6362.20 ± 1227.29		4987.40 ± 477.66

All values are shown as mean ± SEM with an n ≥ 5.

Table S2. pH of recording buffers in the presence of chelating agents.

	**ND96**	**Tricine**		**Ca-EDTA**	
		**1 mM**	**10 mM**	**10 µM**	**100 µM**
**pH**	7.47	7.46	7.47	7.45	7.45

ND96 buffer was prepared using standard methods. From the ND96 solution, solutions containing tricine or Ca-EDTA were made. The pH of solutions were measured using an electric pH meter.
